# Boron-Doped BiOBr Nanosheets with Enhanced Photocatalytic Activity for Sulfanilamide and Dyes

**DOI:** 10.3390/molecules30081735

**Published:** 2025-04-12

**Authors:** Zimu Wei, Ying Wang, Zonghan Shao, Linkun Xie, Lianpeng Zhang, Kaimeng Xu, Xijuan Chai

**Affiliations:** 1 Yunnan Key Laboratory of Wood Adhesive and Glued Products, Southwest Forestry University, Kunming 650224, China; 2 College of Material and Chemical Engineering, Southwest Forestry University, Kunming 650224, China

**Keywords:** BiOBr nanosheets, hydrothermal method, compound material, visible photocatalytic degradation, degrade contaminants

## Abstract

A boron-doped BiOBr photocatalytic nanosheet was synthesized using a one-step hydrothermal method. The effects of solvent, temperature, and boron doping content on the morphology and photocatalytic performance were investigated. The boron-doped samples synthesized with acetic acid at 180 °C (1B-AB) showed optimal photocatalytic performance, achieving 80% efficiency in degrading sulfanilamide (SN) within 6 h. After five cycles, the degradation rate decreased by 21%. The 10% boron doping reduced BiOBr’s bandgap (from 2.90 to 2.88 eV), improving visible light utilization and reducing electron–hole pair recombination. The 1B-AB photocatalyst also demonstrated excellent activity against anionic dyes like methyl orange (MO) and malachite green (MG). Hydroxyl radicals (·OH) and superoxide anions (·O_2_^−^) were identified as the main active species in the SN degradation process.

## 1. Introduction

Sulfonamide antibiotics (SAs) and azo dyes are prevalent organic pollutants in water sources due to their extensive use in the medical and industrial fields [1]. These compounds exhibit high environmental persistence, ecotoxicity, bioaccumulation potential, and resistance to degradation, resulting in their prolonged presence in the environment. Such persistence poses significant risks to both ecosystems and human health [2,3,4]. Therefore, developing efficient and sustainable technologies for the removal of persistent organic pollutants from water has become a critical challenge in the field of environmental pollution remediation [5].

Photocatalytic technology is recognized as an efficient approach for water pollutant removal, owing to its favorable characteristics such as mild reaction conditions, ease of implementation, high efficiency, environmental friendliness, and cost-effectiveness [6]. Research on photocatalysts has primarily focused on the development of various semiconductor materials, including non-metallic photocatalysts, metal sulfides, and metal oxides [7]. As a typical V-VI-VII ternary semiconductor, bismuth oxybromide (BiOBr) has become a research hotspot in the field of photocatalysis due to its remarkable visible light absorption capacity, low toxicity, and excellent photocatalytic efficiency [8]. However, the intrinsic wide bandgap and low specific surface area of pristine BiOBr limit its potential for visible light absorption and photocatalytic degradation [9]. Therefore, improving its photocatalytic performance through morphological and structural tuning, as well as doping strategies, has become a key research challenge.

Compared to bulk materials, nanosheets (NSs) have shorter charge carrier migration distances and a large number of exposed internal atoms, which can reduce the recombination rate of photo-induced charge carriers while increasing the available active sites on the catalyst, thereby significantly enhancing photocatalytic activity. The nanosheet structure of BiOBr consists of alternating positively charged [Bi_2_O_2_]^2+^ layers and negatively charged halide ion layers. This unique structure facilitates effective separation of photo-generated charge carriers and doping modifications [10]. Shen et al. synthesized BiOBr nanosheets with a thickness of approximately 60 nm in a dilute HNO_3_ hydrothermal environment [11]. Wang et al. dissolved Bi(NO_3_)_3_·5H_2_O in octadecene in the presence of oleic acid and oleylamine, and then introduced a KBr aqueous solution containing HNO_3_ to prepare BiOBr nanosheets [12]. Li et al. prepared BiOBr nanosheets with a thickness of about 100 nm in a mannitol hydrothermal environment [13]. Compared to the highly corrosive and expensive organic solvents mentioned above, the nanosheets synthesized by Zhang et al. in an acetic acid aqueous environment exhibit significantly better environmental friendliness [14]. However, a review of previous studies reveals a lack of systematic research on the effects of mild hydrothermal environments on the structure and performance of BiOBr nanosheets.

Element doping is a straightforward and effective approach to modulating the structure of photocatalysts. Typically, a small amount of dopant can significantly alter the electronic structure of the catalyst, creating new impurity energy levels that narrow the band gap and enhance light absorption. Compared to metal doping, non-metal doping offers advantages such as lower cost, reduced susceptibility to photo-corrosion, and greater environmental friendliness [15,16]. Among various non-metal elements, boron (B) is characterized by its light weight, small atomic radius, stability, and ability to diffuse into semiconductor lattices [17,18]. Its vacant p-orbitals can interact with the p-orbitals of oxygen (O) in oxygen-containing catalysts, potentially leading to a reduction in the band gap [19]. Fu et al. prepared Bi_2_WO_6_-B composite materials using an in situ hydrothermal reduction method. By utilizing rhodamine B (RhB) as the target for degradation, the extent of photocatalytic degradation for Bi_2_WO_6_-B under visible light was observed to be 2.4 times greater than that of Bi_2_WO_6_ [20]. The ionic radius of boron is 0.23 Å, significantly smaller than that of bismuth (Bi), which is 1.03 Å. Therefore, boron can be easily incorporated into the BiOBr lattice to modulate its band structure [21]. Shen et al. synthesized boron-doped bismuth oxybromide (B-doped BiOBr) for CO_2_ photoreduction. The study found that boron doping extended the light absorption range of BiOBr and enhanced the separation efficiency of photogenerated charge carriers [11]. Wu et al. synthesized B-doped BiOBr for photocatalytic inactivation of *Escherichia coli*. Under visible light, it exhibited significantly higher antibacterial activity against *E. coli* K-12 compared to pure BiOBr nanosheets [21]. Therefore, introducing boron (B) atoms with electron-deficient characteristics into the BiOBr lattice can significantly alter the electronic structure of the catalyst and promote the separation of photogenerated charge carriers.

As aforementioned, antibiotics and dyes, particularly mixed dyes, pose serious threats to human health and the ecological environment. Unfortunately, research on the photocatalytic degradation of antibiotics and mixed dyes using B-doped BiOBr is still limited. In this study, BiOBr nanosheets with varying boron doping ratios were synthesized via a one-step method in a mild hydrothermal environment. The effects of different hydrothermal solvents, hydrothermal temperatures, and boron doping molar ratios on the structure, electronic distribution, and photocatalytic degradation performance of BiOBr were systematically investigated. The enhancement mechanism of boron doping on the photocatalytic performance of BiOBr was revealed, and its application in the degradation of sulfonamide antibiotics and azo dye mixtures was explored. The research provides valuable insights for the development of efficient and stable doped BiOBr photocatalysts in mild hydrothermal environments and practical application in the degradation of persistent pollutants.

## 2. Results and Discussion

### 2.1. Microstructure and Chemical Composition

**Figure 1:**
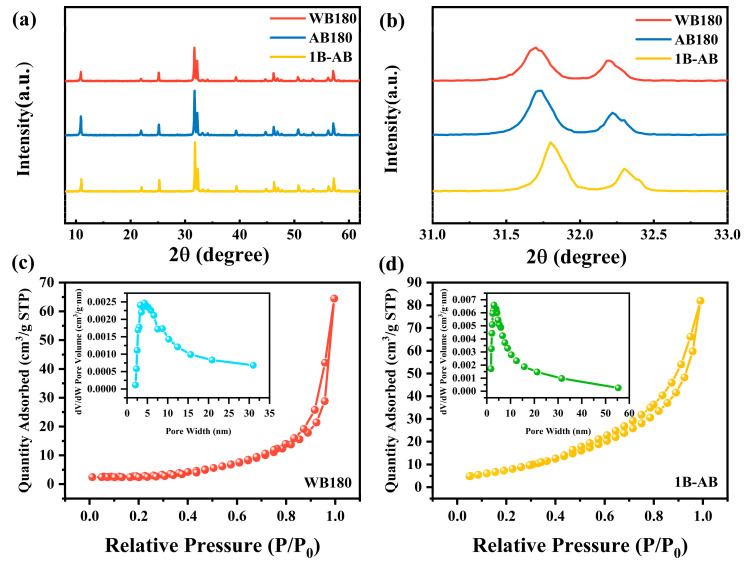
(**a**,**b**) X-ray diffraction (XRD) and (**c**,**d**) N_2_ adsorption–desorption isotherms and pore size distribution images of the prepared samples.

X-ray diffraction (XRD) was used to analyze the phase composition and crystallinity of the samples. As illustrated in Figure 1a, the pure BiOBr sample (AB180), synthesized in an acetic acid environment, exhibited a stronger diffraction intensity compared to the BiOBr sample (WB180) prepared in pure water. Notably, no impurity phases were detected in the diffraction peaks of any of the samples, indicating that boron (B) doping did not alter the phase structure. With the introduction of B doping, the positions of the (012) and (110) peaks gradually shifted to higher angles, as shown in Figure 1b. Bragg’s law states that an increase in the 2θ value indicates a decrease in lattice parameters [22]. Typically, boron (B) dopants can be categorized into two modes: interstitial doping and substitutional doping. The former mode tends to increase the lattice parameters, whereas the latter may result in variable changes in the lattice parameters, contingent upon the differences in ionic radii [23]. B^3+^ has a much smaller ionic radius (23 pm) compared to Bi^3+^ (96 pm), the observed shift in the diffraction peaks may suggest that boron ions are incorporated into the BiOBr lattice through the substitution of Bi^3+^ ions.

As shown in Figure 1c,d, the adsorption–desorption curves of WB180 and 1B-AB (corresponding to B/Bi molar ratios of 10%) exhibit type IV isotherms with H3-type hysteresis loops. The type IV isotherm indicates weak interactions between the material and nitrogen [24]. Additionally, a higher relative pressure at which capillary condensation occurs suggests a larger pore size. From the figure, it is evident that WB180 exhibits capillary condensation at a higher relative pressure compared to 1B-AB, indicating that the former has a larger pore size, which is consistent with the pore size data in Table S1. According to Table S1, the specific surface area of WB180 is only 9.726 m^2^/g, whereas that of 1B-AB reaches 31.408 m^2^/g. This indicates that the structure formed by doping BiOBr with boron (B) effectively increases its specific surface area. Generally, a larger specific surface area of photocatalyst materials not only enhances their contact area with organic pollutants but also improves the adsorption and transport of reactants, thereby significantly boosting degradation efficiency. Therefore, 1B-AB is expected to exhibit superior photocatalytic performance.

**Figure 2:**
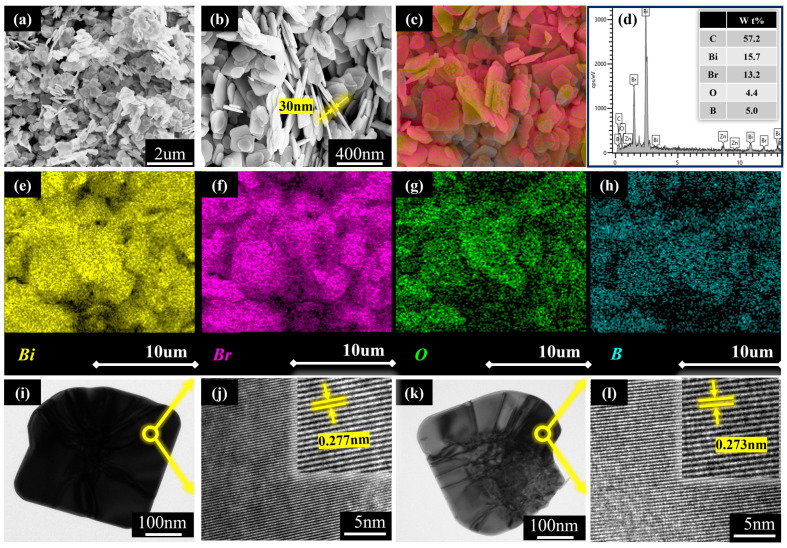
SEM and EDS images of 1B-AB catalyst (**a**–**h**); TEM images of WB180 catalyst (**i**) and its corresponding magnified view (**j**); TEM images of 1B-AB catalyst (**k**) and its corresponding magnified view (**l**).

The scanning electron microscope (SEM) is an important instrument for analyzing the microstructure of the produced materials. Figure 2a–c present SEM images of Bi/BiOBr nanosheets, which reveal that BiOBr has a thickness of approximately 30 nm, with relatively regular shapes and good dispersion. This configuration results in a layered stacked nanostructure with a smooth surface. The hierarchical nanoflower architecture indicates an increased specific surface area and a higher number of active sites for photocatalytic processes, thereby improving both adsorption and photodegradation efficiency. Furthermore, EDS mapping was employed to elucidate the elemental dissemination within the B-doped BiOBr nanosheets. The findings, illustrated in Figure 2c–h, demonstrate that the elements (Bi, O, Br) are uniformly distributed throughout the composite nanosheets. Additionally, as depicted in Figure 2i, the analysis confirms that the precise content of B doped in 1B-AB is 5 wt%, thereby verifying the successful incorporation of boron atoms into BiOBr, with no other elements or impurities detected.

To further investigate the surface state and lattice structure of the synthesized materials, transmission electron microscopy (TEM) characterization was performed, with the results presented in Figure 2i–l. The boron-doped ultrathin BiOBr samples displayed distinct lattice fringes. In Figure 2j,l, the measured lattice fringe distance of 0.277 nm and 0.273 nm aligns with the (102) and (110) crystalline planes of the tetrahydroxide. This finding indicates that the synthesized BiOBr nanosheets exhibit a highly exposed (102) crystal plane and demonstrate commendable crystallinity along the crystal belt axis direction.

**Figure 3:**
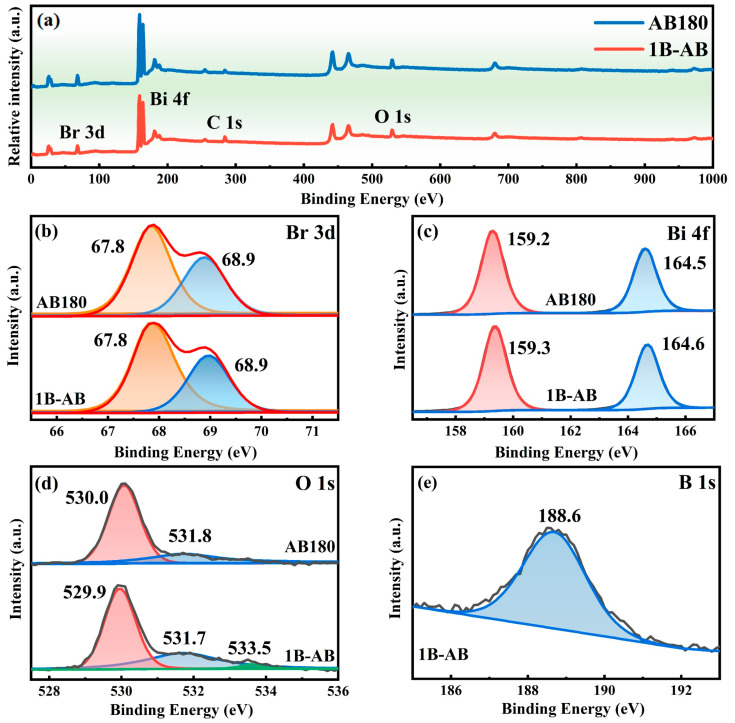
XPS for AB180 and 1B-AB (**a**); Br 3d (**b**); Bi 4f (**c**); O 1s (**d**); and B 1s (**e**) spectra in 1B-AB.

The 1B-AB catalyst’s chemical composition was analyzed using X-ray photoelectron spectroscopy (XPS), calibrated to the C 1s peak at 284.8 eV. The measured spectrum (Figure 3a) reveals the presence of bismuth (Bi), bromine (Br), and oxygen (O) elements in both the AB180 and 1B-AB samples. Nonetheless, the boron (B) signal was missing in the 1B-AB specimen, probably because of the low doping levels and the significant dispersion of B atoms. In contrast, the presence of B doping has been confirmed through energy-dispersive spectroscopy (EDS) mapping and elemental analysis.

In Figure 3b, the two signals detected at 67.8 eV and 68.9 eV, relating to the Br 3d region, are assigned to the Br 3d_5/2_ state in 1B-AB, thereby confirming the presence of bromine. In Figure 3c, the peaks located at 159.2 eV and 164.5 eV in the Bi 4f region can be credited to the Bi 4f_5/2_ and Bi 4f_7/2_ states in 1B-AB, which are indicative of Bi^3+^ in BiOBr [25]. The binding energies of the Bi 4f and Br 3d spectra for the two samples exhibited no significant variations, suggesting that the incorporation of boron did not influence the electron density surrounding bismuth. This observation may be attributed to the comparable electronegativities of boron (2.04) and bismuth (2.02).

Figure 3d presents the O 1s spectrum of the boron-doped BiOBr catalyst. Two distinct peaks are identified at 530.0 eV and 531.8 eV, corresponding to lattice oxygen and hydroxyl oxygen, respectively. A faint absorption feature at 533.5 eV in the O 1s spectrum of the 1B-AB specimen suggests the existence of a B-O bond [26]. The findings indicate that the comparable electronegativity values of boron (B) at 2.04 and bismuth (Bi) at 2.02 result in negligible alterations in the displacement of the Bi-O bond. Consequently, the incorporation of boron as a dopant is unlikely to influence the electron density surrounding the oxygen (O) atoms, nor will it alter the van der Waals forces between the layers in the BiOBr crystal lattice.

Figure 3e presents the B 1s spectrum of the 1B-AB sample. The spectral region of B 1s displays notable asymmetrical characteristics due to its overlap with the Br 3p_1/2_ peak. The binding energy value is situated between the molecular binding energy of B 1s (187.3 eV) and the binding energy of B 1s in the gap mode (192.6 eV), suggesting the presence of at least one chemical valence state of boron in the 1B-AB sample. The peak at 188.6 eV corresponds to the B-O bond, but no XPS peak for B_2_O_3_ was found at 192.4 eV [27]. The findings suggest that the 1B-AB sample does not contain crystalline B_2_O_3_ or H_3_BO_3_ substances. This further supports the conclusion that boron (B) has been incorporated into the lattice structure of BiOBr, likely in a substitutional manner as B^3+^ ions, rather than existing as a mere physical mixture.

According to the previously mentioned XPS analysis, boron-doped BiOBr nanosheet photocatalysts were synthesized. It is hypothesized that the boron (B) atoms substitute for bismuth (Bi) in the crystal lattice and are incorporated into the [Bi_2_O_2_] units of the BiOBr structure. This structure efficiently analyzes the effect of boron doping on the structure and photocatalytic performance of BiOBr.

### 2.2. Photocatalytic Performance

**Figure 4:**
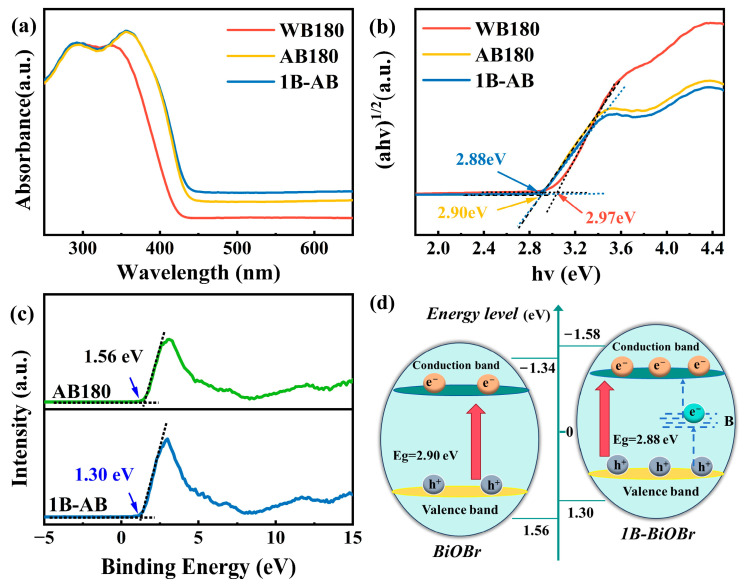
(**a**) UV–vis DRS spectra of WB180, AB180, and 1B-AB samples; (**b**) corresponding band gap Tauc diagram; (**c**) VB-XPS spectra of AB180 and 1B-AB; (**d**) 1B-AB bandgap structure.

The optical properties of the specimens were assessed using UV–visible diffuse reflectance spectroscopy within a wavelength range of 250 to 650 nm. As shown in Figure 4a, a significant redshift in the absorption boundary of 1B-AB was noted in contrast to AB180. This observation implies that the inclusion of B-doped atoms greatly enhances the capacity to absorb visible light [28]. Similarly, for WB180, the more compact layered configuration, which aligns with the band edges of the semiconductor, causes a marked redshift in the adsorption boundary of AB180.

The Tauc plot method (Figure 4b) was used to determine the band gap values (Eg) of the photocatalysts by converting the UV–vis DRS spectra into (ahv)^2^-hv plots. The calculated band gap values for WB180, AB180, and 1B-AB are 2.97 eV, 2.90 eV, and 2.88 eV, respectively. These findings indicate that both boron (B) doping and the formation of layered structures contribute positively to the reduction in Eg and the enhancement of visible light absorption capabilities [29]. The smaller atomic radius and higher electronegativity of boron could alter the local electronic environment around the Bi and O atoms in BiOBr, causing a reduction in the valence band energy level, thus leading to a decrease in the overall band gap energy [21]. Therefore, the position of the valence band for the samples was ascertained using valence band X-ray photoelectron spectroscopy (VB-XPS). As illustrated in Figure 4c, the valence band positions of AB180 and 1B-AB were measured to be 1.56 eV and 1.30 eV, respectively. Utilizing the equation (E_CB_ = E_VB_ − E_g_), E_CB_ edges for AB180 and 1B-AB can also be calculated, yielding values of −1.319 eV and −1.342 eV, respectively [30].

Building on the previously mentioned findings, Figure 4d elucidates the photocatalytic degradation mechanism of 1B-AB. Initially, the organic compound sulfanilamide is adsorbed onto the surface of the photocatalyst, which is subsequently followed by the photocatalytic reaction. Furthermore, the introduction of impurity levels between CB and VB serves to reduce the band gap of the BiOBr photocatalyst. Concurrently, oxygen vacancies function as electron traps, thereby facilitating charge transport. The findings indicate that 1B-AB possesses the narrowest bandgap and exhibits the highest sensitivity to visible light. Absorption of energy from visible light excites electrons in the valence band (VB) to the conduction band (CB) of BiOBr or to oxygen vacancy states, generating holes in the valence band. During the photocatalytic reaction, the excited electrons preferentially migrate to the impurity B and oxygen vacancies, rather than recombining with the remaining holes, thus boosting the effective separation of light-induced electron–hole pairs. Additionally, the B element deposited on the surface of BiOBr can be activated by visible light irradiation throughout the wavelength spectrum of 410–870 nm, causing the formation of extra electrons.

### 2.3. Photocatalytic Measurements

**Figure 5:**
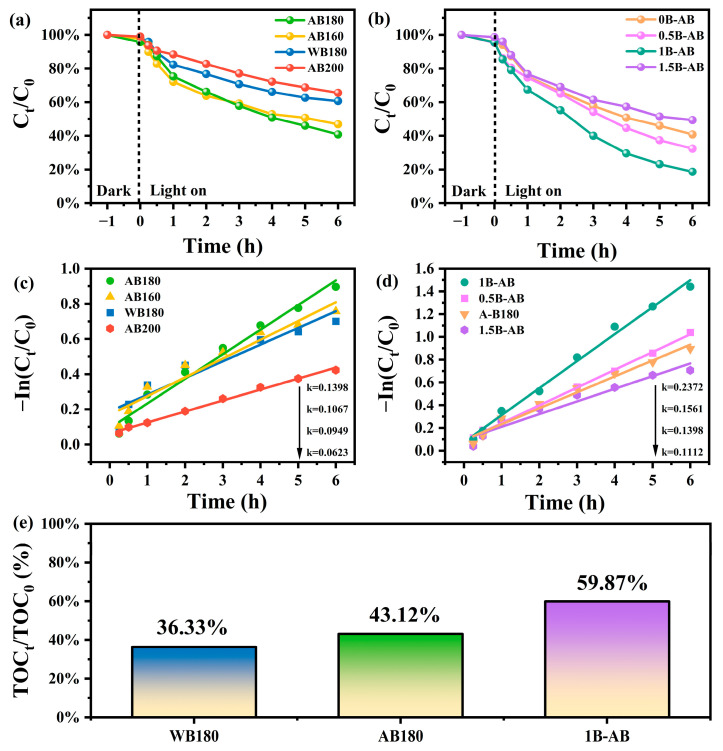
Adsorption-photocatalytic performance of sulfonamides (10 mg/L) on the modified BiOBr catalyst (**a**,**b**) and the corresponding photocatalytic degradation rate (**c**,**d**) and TOC removal rate results in the sulfonamides degradation experiment (**e**).

Different hydrothermal temperatures and varying levels of boron doping significantly influence the photocatalytic activity of BiOBr. In this study, sulfonamide was identified as the optimal dye for selective adsorption. The adsorption process commences with a dark adsorption phase lasting one hour, during which the system gradually approaches adsorption equilibrium, subsequently followed by the photocatalytic degradation of sulfonamide-contaminated wastewater. The adsorption capacity and the associated mechanisms of the modified BiOBr catalyst were thoroughly analyzed. Figure 5a illustrates the impact of varying temperatures on the adsorption capacity of sulfonamide onto BiOBr samples. Notably, for the modified BiOBr samples, the removal rate of sulfonamide at a hydrothermal temperature of 180 °C can reach 60% as the temperature is adjusted. Figure 5c examines the reaction kinetics of sulfonamide degradation on the modified catalyst. The degradation mechanism follows pseudo-first-order kinetics, with the calculated apparent rate constant (k) for the AB180 sample determined to be 0.1398 min^−1^, which surpasses the degradation rate of the WB180 sample (k = 0.0949 min^−1^). This further underscores the enhanced photocatalytic performance of the AB180 catalyst [31].

The doping concentration of boron (B) in bismuth oxybromide (BiOBr) crystals significantly influences the photocatalytic activity of the samples. As illustrated in Figure 5b, after six hours of ultraviolet (UV) light irradiation, the removal rates of sulfonamides for the samples designated as 0.5B-AB, 1B-AB, and 1.5B-AB were recorded at 35%, 24%, and 49%, respectively. As depicted in Figure 5d, an increase in B doping content from 0.05 wt% (0.5B-AB) to 0.1 wt% (1B-AB) resulted in a gradual enhancement of photocatalytic degradation efficiency, with the 1B-AB sample exhibiting the highest degradation efficiency (k = 2372 min^−1^). Conversely, when the B content was further increased from 0.1 wt% (1B-AB) to 0.15 wt% (1.5B-AB), a decline in photocatalytic activity was observed. Prior research has demonstrated that non-metallic elements employed as doping agents can significantly impede the recombination of photogenerated electron–hole pairs [32]. Excessive doping content can hinder photocatalytic activity, primarily due to the potential formation of lattice defects associated with excessive boron (B) doping. These defects serve as recombination centers for photogenerated charge carriers, consequently diminishing photocatalytic efficiency. In contrast, doping with 0.4 mmol of H_3_BO_3_ enhances the photocatalytic performance of 1B-AB. This finding indicates that an ideal concentration of H_3_BO_3_ enhances the dissociation of photogenerated electron–hole pairs while concurrently reducing their recombination.

Moreover, the total organic carbon (TOC) values were measured to determine the mineralization rate of sulfadiazine in the photocatalytic system. As shown in Figure 5e, the mineralization efficiencies of WB180, AB180, and 1B-AB were 36.33%, 43.12%, and 59.87%, respectively. These results indicate that the sulfadiazine molecular chains were effectively decomposed into small molecules and carbon dioxide through the mineralization process.

### 2.4. Degradation Dye Test

**Figure 6:**
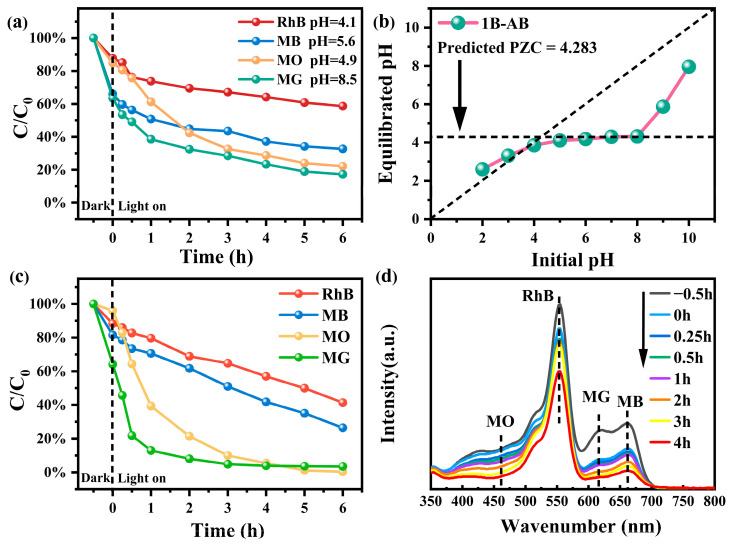
Time process of adsorption photodegradation of single dye (**a**); the point of zero charge (PZC) of 1B-AB (**b**); multi-component mixed dye (**c**) on MO, MG, MB, and RhB (20 mg/L) using catalyst 1B-AB; (**d**) UV degradation spectra under multiple dyes.

Before investigating the photocatalytic degradation of dyes, the adsorption behavior of the 1B-AB catalyst toward different ionic dyes was systematically examined, particularly the anionic dye methyl orange (MO) and the cationic dyes malachite green (MG), rhodamine B (RhB), and methylene blue (MB). As shown in Figure 6a, in a single-dye system, 1B-AB exhibited a high adsorption capacity for MG and MB within 30 min of dark adsorption, achieving removal rates of 36% and 34%, respectively. In contrast, the adsorption efficiencies for MO and RhB were relatively lower, at 15% and 13%, respectively. During the subsequent photocatalytic degradation process, the degradation rate of MO increased significantly, with the final degradation efficiency following the order MG (83%) > MO (78%) > MB (67%) > RhB (41%).

Based on Figure 6b, the zero-point charge pH_PZC_ of 1B-AB was estimated to be 4.283 (while the recent literature reports that the point of zero charge for BiOBr is 5.30 [33,34]). When pH < pH_PZC_, the catalyst surface was positively charged, favoring the electrostatic adsorption of anionic dyes. Conversely, when pH > pH_PZC_, the catalyst surface became negatively charged, leading to electrostatic repulsion of anionic dyes. Furthermore, pH measurements of different dye solutions revealed that MG exhibited weak alkalinity, whereas the other dye solutions were weakly acidic. As a result, the cationic dye MG could stably adsorb onto the catalyst surface and undergo rapid degradation under photocatalytic conditions. Additionally, MG and MB molecules are rich in conjugated π-electrons, which can interact more effectively with photogenerated electron–hole pairs during the photocatalytic degradation process, thereby facilitating the degradation reaction. In contrast, RhB has a more stable molecular structure, and its degradation may involve a stepwise deethylation process, which proceeds more slowly. Consequently, RhB exhibited the lowest final degradation rate (41%).

In the multi-dye system in Figure 6c, the 1B-AB catalyst significantly improves the removal efficiency of different dyes [35]. The observed phenomenon may be attributed to the ionic interactions among multiple dyes, which enhance the mutual adsorption reactions between the various dyes and subsequently improve degradation efficiency. Nevertheless, the degradation efficiency of rhodamine B (RhB) remains suboptimal, potentially due to the large molecular structure of the RhB dye, which obstructs the effective adsorption pathways of the catalyst 1B-AB. This obstruction diminishes the effective photocatalytic contact area with the catalyst, thereby impeding the removal efficiency of RhB.

To provide a more intuitive illustration, Figure 6d presents the UV–visible spectral curves of the mixed dye solution throughout the degradation process. The absorption peaks at 464 nm, 554 nm, 617 nm, and 664 nm correspond to MO, MB, MG, and RhB. Following exposure to visible light, the absorption peaks of MO and MG exhibit a rapid decline. In contrast, the UV–visible spectrum of RhB continues to display a distinct characteristic peak within the visible light region at 554 nm [36]. The degradation rate of rhodamine B is notably slow, as evidenced by the substantial amount of residual rhodamine present after 5 h of reaction. This observation suggests that the 1B-AB catalyst exhibits a superior degradation capacity for methyl orange (MO) and malachite green (MG) in comparison to rhodamine B (RhB), which aligns with the degradation effects observed for the individual dyes previously discussed. The findings indicate that the synthesized 1B-AB catalyst possesses a specific surface area and pore structure that are conducive to its function and that the molecular weight of various dye solutions significantly influences the adsorption behavior of the 1B-AB catalyst.

### 2.5. Charge Transfer Mechanism

**Figure 7:**
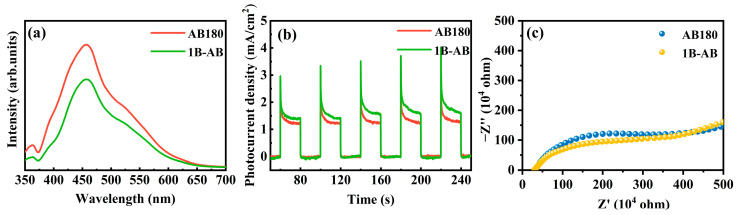
(**a**) Photoluminescence spectrum of the sample; (**b**) photocurrent response; and (**c**) EIS impedance spectrum.

To investigate the behavior of light-induced charge transfer, photoluminescence (PL) and photoelectrochemical analyses were performed on the synthesized samples. As shown in Figure 7a, the fluorescence intensity of 1B-AB is significantly lower compared to that of AB180 and 1B-AB. This observation can be ascribed to the existence of boron-doped BiOBr, which suppresses charge carrier recombination, enhances electron capture, and improves the utilization of both electrons and holes during the redox process. Consequently, this effectively extends the charge lifetime within the catalyst [37], thereby enhancing its degradation activity.

Further investigations used photoelectric current measurements and electrochemical impedance spectroscopy to evaluate photogenerated charge separation efficiency. Figure 7b shows the temporary photoelectric current response of the catalyst when exposed to visible light illumination. Among the samples analyzed, the photoelectric current of AB180 was found to be lower than that of 1B-AB. This observation suggests that the defect effects induced by B doping facilitate reduced recombination rates and promote more rapid charge transfer [38].

Moreover, analogous results were observed in the electrochemical impedance spectroscopy (EIS) analysis of the samples (Figure 7c). Typically, a smaller radius of the Nyquist semicircle in EIS signifies an enhanced charge transfer capability, which mitigates the recombination of photogenerated carriers. In comparison to AB180, the 1B-AB sample exhibits a diminished radius of the Nyquist curve [39]. In conclusion, boron doping of ultrathin BiOBr enhances the separation efficiency of photogenerated electrons and holes (*e^−^* − *h^+^*), which is advantageous for enhancing photocatalytic activity. This enhancement is the underlying mechanism contributing to the remarkable photocatalytic performance of 1B-AB nanosheets.

**Figure 8:**
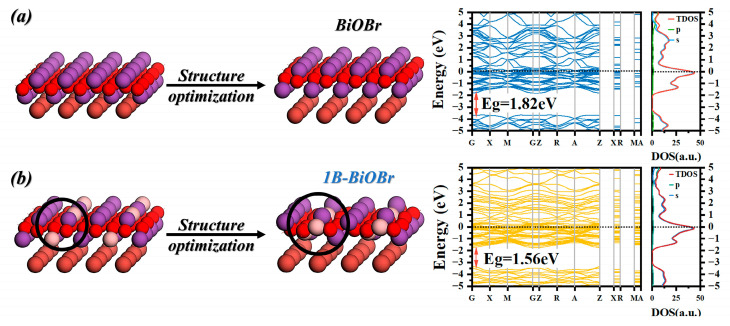
The structure, optimized structure, calculated band structure, and density of states (DOS) of (**a**) BiOBr and (**b**) 1B-AB (001) surfaces.

The electronic band structure and density of states (DOS) of BiOBr and 1B-AB were investigated through theoretical calculations. Figure 8 presents the possible structures of BiOBr and 1B-AB used for the calculations, along with their corresponding band structures. Due to the inherent limitations of the computational methods, the calculated band gaps of BiOBr and 1B-AB are relatively narrow. Compared to BiOBr (Eg = 1.82 eV, Figure 8a), 1B-AB exhibits a reduced band gap (1.56 eV, Figure 8b). Additionally, boron doping introduces significant donor levels, and the presence of defect states forms an intermediate step between the valence band (VB) and the conduction band (CB), allowing electrons to gradually transition from VB to CB. This enhances the material’s visible-light absorption. The computational results are consistent with the predictions from XPS and UV–vis analyses.

### 2.6. Free Radical Species Capture and Recycling Experiment

**Figure 9:**
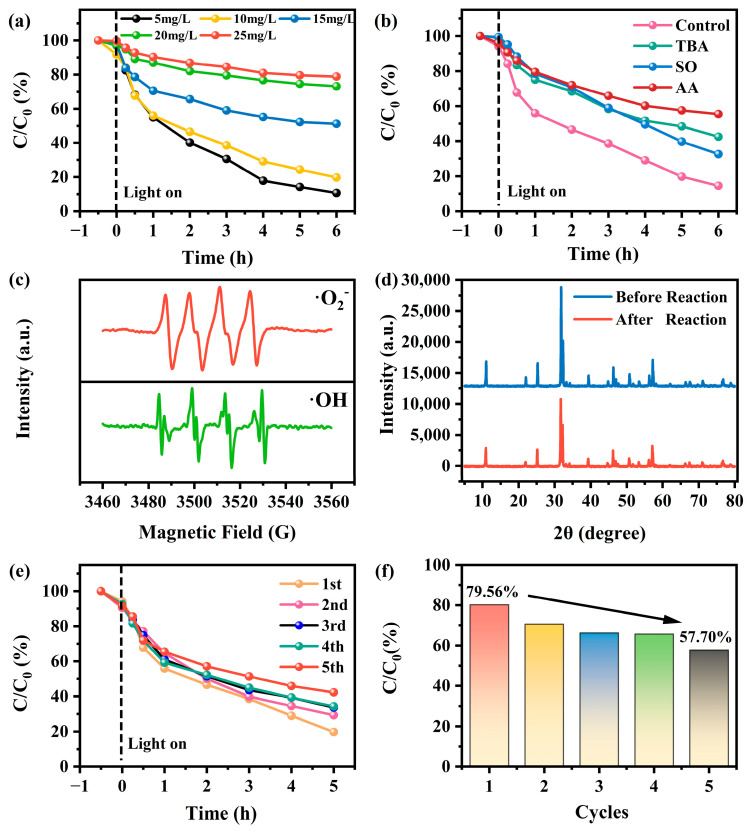
Photocatalytic degradation of different concentrations of sulfonamide (**a**) and capture experiments of active species using catalyst 1B-AB (**b**); DMPO-·O_2_^−^ and -·OH EPR Spectra for 1B-AB (**c**); XRD patterns of the sample before and after reaction (**d**); cyclic experiments (**e**,**f**) on the degradation of sulfonamide (10 mg/L) by 1B-AB.

Figure 9a illustrates the impact of varying initial concentrations of sulfonamides on the photodegradation system utilizing 1B-AB. The findings indicate that photocatalytic efficiency is maximized at a concentration of 10 mg/L, whereas elevated concentrations impede the degradation process. These findings indicate a significant relationship between the adsorption process and the starting concentration, with the produced samples showing improved effectiveness for the photodegradation of dye solutions at reduced concentrations.

As shown in Figure 9b, the photodegradation rate of sulfonamides reached 86% in the absence of scavengers, indicating the high degradation efficiency of the system. However, the introduction of scavengers such as tert-butanol (TBA), sodium oxalate (SO), and ascorbic acid (AA) resulted in a significant decrease in the photodegradation efficiency, suggesting the involvement of multiple reactive species in the reaction process. Specifically, after 6 h of reaction, the removal rates of sulfonamides decreased to 57.8% (with TBA), 68.2% (with SO), and 45.4% (with AA), respectively [40]. Furthermore, electron paramagnetic resonance (EPR) analysis of the generated radical signals after 5 min of illumination revealed distinct characteristic peaks of DMPO-·O_2_^−^ and DMPO-·OH (Figure 9c). This confirms the participation of ·OH and ·O_2_^−^ in the photocatalytic degradation process, which is consistent with the results of the radical scavenging experiments. Consequently, the high removal efficiency of sulfonamides can be attributed to the active species ·OH and ·O_2_^−^ generated by the application of 1B-AB under UV light irradiation.

Moreover, it is essential to consider the recyclability and physicochemical stability of the 1B-AB photocatalyst during the degradation of sulfonamides. As shown in Figure 9d, the XRD patterns of the catalyst before and after the reaction show no significant changes, indicating the structural stability of the sample. Cyclic tests further evaluated the degradation efficiency of sulfonamides. The results are presented in Figure 9e,f. After five cycles, the 1B-AB photocatalyst maintained its ability to decompose sulfonamides, achieving a degradation efficiency of 57.7%. There is a certain degree of performance degradation in the sample, which may be attributed to sample loss during the collection and decontamination processes [41]. Thus, the 1B-AB photocatalyst demonstrates a notable degree of reusability and stability in practical applications. The analysis shows that the 1B-AB photocatalyst is an effective and stable nanomaterial for visible light applications.

Table 1 further validates the exceptional performance of 1B-AB. Compared to BiOBr-based catalysts developed over the past five years, it requires relatively lower dosage while exhibiting superior multifunctional degradation capability and broad applicability under low-power cold light sources.

### 2.7. Photocatalytic Mechanism

**Figure 10:**
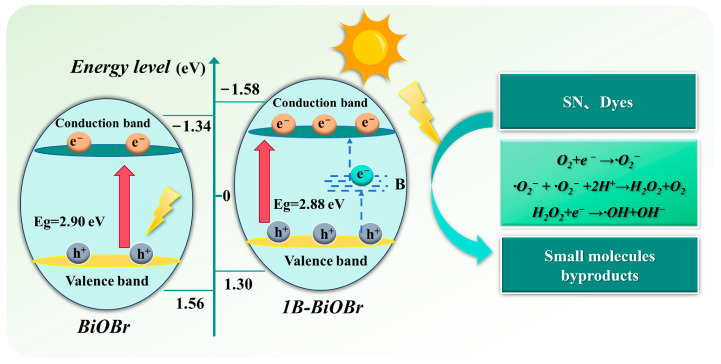
Schematic diagram of photocatalytic degradation of pollutants by 1B-AB.

Figure 10 illustrates the degradation mechanism associated with the removal of dyes and sulfonamides by the 1B-AB sample under visible light conditions. In both acidic and neutral environments, BiOBr is capable of forming two-dimensional layered nanosheets characterized by high crystallinity along the (012) crystal belt axis. These nanosheet structural units facilitate the adsorption of dyes and sulfonamides from the reaction system onto the (012) crystal plane of B-doped BiOBr via a diffusion process. Given that boron does not react with halogen ions in neutral and acidic media, it is incorporated into the BiOBr lattice through a hydrolysis method. EDS and XPS analyses confirm that the successful incorporation of boron enhances the presence of surface hydroxyl groups. This enhancement not only increases the adsorption capacity for dye molecules on the photocatalyst but also enhances the separation of charge carriers produced under light exposure, thus increasing the photocatalytic performance of the boron-doped BiOBr sample. UV–vis spectroscopy indicates that boron introduction broadens the absorption range, allowing for greater photon absorption in the visible spectrum and improving the sample’s visible light absorption ability.

Under visible light, the valence band electrons of boron-doped bismuth oxybromide (B-doped BiOBr) are excited to a lower energy level. In this context, boron serves as an electron trap, capturing electrons (e-) from the valence band (VB) and reducing recombination in the conduction band (CB) of BiOBr. Subsequently, the dissolved oxygen adsorbed on the surface of the semiconductor interacts with the electrons in the CB to generate hydroxyl radicals (·OH) and superoxide anions (·O_2_^−^). Simultaneously, the holes in the valence band of BiOBr interact with water (H_2_O) to generate ·OH radicals. Consequently, the ·OH and ·O_2_^−^ species generated by the photogenerated electron–hole pairs can directly oxidize large molecular dyes and other organic substances, facilitating the desorption of degradation products from the catalyst surface and their diffusion into the liquid phase. The band gap (Eg) of boron-doped BiOBr and unmodified BiOBr, measured using UV–vis diffuse reflectance spectroscopy (DRS), is 2.88 eV and 2.90 eV, respectively. A smaller band gap in B-doped BiOBr correlates with an increased electron transfer rate and enhanced photocatalytic activity.

## 3. Materials and Methods

### 3.1. Materials

Sodium bromide (NaBr, Analytical Reagent (AR), China National Pharmaceutical Group, Beijing, China), glacial acetic acid (CH_3_COOH, AR, China National Pharmaceutical Group, Beijing, China), bismuth nitrate pentahydrate (Bi(NO_3_)_3_·5H_2_O, AR, Tianjin Chemical Reagent Third Factory, Tianjin, China), boric acid (H_3_BO_3_, AR, China National Pharmaceutical Group, Beijing, China), tert-butanol (TBA, AR, China National Pharmaceutical Group, Beijing, China), sodium oxalate (SO, China National Pharmaceutical Group, Beijing, China), ascorbic acid (AA, AR, China National Pharmaceutical Group, Beijing, China), sulfanilamide (SN, AR, China National Pharmaceutical Group, Beijing, China), rhodamine B (RhB, AR, China National Pharmaceutical Group, Beijing, China), methylene blue (MB, AR, Tianjin Chemical Reagent Third Factory, Tianjin, China), methyl orange (MO, AR, China National Pharmaceutical Group, Beijing, China), and malachite green (MG, AR, China National Pharmaceutical Group, Beijing, China).

### 3.2. Preparation of BiOBr Nanosheets

An amount of 4 mmol of NaBr was dissolved in 30 mL of aqueous acetic acid solution, maintaining a volume ratio of acetic acid to water of 1:2. Subsequently, 50 mL of Bi(NO_3_)_3_·5H_2_O aqueous solution, containing 4 mmol of bismuth, was added dropwise to the aforementioned solution. The mixture was stirred at room temperature for 30 min to facilitate the formation of a precursor solution. The precursor solution was then placed in a hydrothermal autoclave and reacted at 180 °C for 12 h. Upon cooling to room temperature, the resultant sample was extracted and rinsed several times with purified water. Finally, the specimen was heated at 60 °C to produce the ultrathin BiOBr specimen, labeled as AB180. In a comparative experiment, additional samples were synthesized at varying temperatures of 160 °C and 200 °C named as AB160 and AB200. At 180 °C, BiOBr nanosheets were produced under identical process conditions in a pure water environment, designated as WB180.

### 3.3. Preparation of B-Doped BiOBr Nanosheets

**Figure 11:**
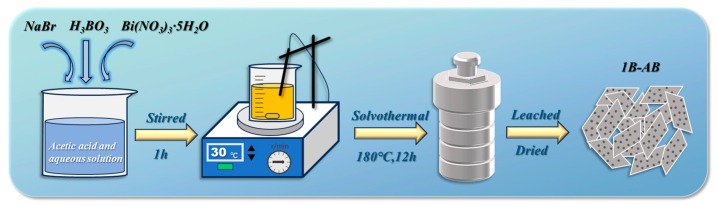
Schematic diagram of the preparation process for 1B-AB materials.

An amount of 4 mmol of NaBr and a specified quantity of H_3_BO_3_ were dissolved in 30 mL of aqueous solution of glacial acetic acid. Following the same procedure, B-doped BiOBr samples were synthesized at a temperature of 180 °C. By varying the amounts of H_3_BO_3_ used, samples with boron to bismuth (B/Bi) molar ratios of 5%, 10%, and 15% were produced, designated as 0.5B-AB, 1B-AB, and 1.5B-AB, respectively. Figure 11 illustrates the preparation process of 1B-AB.

### 3.4. Characterizations

An X-ray diffractometer (XRD, Rigaku Miniflex 600, Tokyo, Japan, Cu, Kα, λ = 1.5406 Å) was used to examine the crystalline phase configuration of the substance, utilizing a 2θ scanning range of 5 to 80 degrees. A field emission scanning electron microscope (SEM, SU-70, Hitachi, Tokyo, Japan) was employed to investigate the three-dimensional microstructure of the substance, and an energy dispersive spectrometer (EDS) attached to the SEM was used for compositional characterization of the samples. A transmission electron microscope (TEM, Tecnai G2 TF-30, Hitachi, Tokyo, Japan) was employed to observe the micro-morphology and perform compositional analysis of the composite materials. An in situ Fourier-transform infrared spectrometer (FT-IR, Great10, Ruijie, Beijing, China) was used to analyze the molecular structural positions and strengths of different functional groups in the material through chemical bond vibrations at absorbance wavelengths of 500–4000 cm^−1^. A 7404 vibrating sample magnetometer (VSM, Lake Shore 7404, Westerville, OH, USA) was utilized to measure the hysteresis curve of the magnetic substances to evaluate the magnetic separation capability of the specimens, with a magnetic field range of ±2 T. An X-ray photoelectron spectrometer (XPS, K-Alpha+, TMO, Waltham, MA, USA) was used to analyze the composite materials before and after the reaction. A UV/visible/near-infrared diffuse reflectance spectrometer (UV–vis, Shimadzu, UV-3600i Plus, Kyoto, Japan) was used to test the light absorption properties of the semiconductor. A photoluminescence testing spectrometer (PL, Fluorolog 3-21, Hitachi, Tokyo, Japan) was used to test the fluorescence intensity of the materials. The mineralization rate was evaluated by measuring the total organic carbon (TOC) content after the reaction using a vario TOC analyzer (TOC, Elementar Vario TOC, Langenselbold, Germany). The specific surface area and pore structure of the materials were characterized using a fully automated specific surface area and pore size analyzer (BET, ASAP-2020, Quantachrome Instruments, Boynton Beach, FL, USA). The specific surface area was determined by the Brunauer–Emmett–Teller (BET) method, and the pore size distribution was calculated using the Barrett–Joyner–Halenda (BJH) method. Nitrogen (N_2_) was used as the adsorbate at a measurement temperature of 77 K. The pore size distribution was calculated in the relative pressure range of P/P_0_ = 0.4–0.98.

### 3.5. Photoelectrochemical Test

The examination of the optoelectronic characteristics of the specimens was performed using an electrochemical workstation (CHI660B, Chenhua, Shanghai, China) configured with a standard three-electrode system. The working electrode comprised an ITO electrode, while a platinum plate acted as the counter electrode, and a saturated Ag/AgCl electrode (with KCl) was employed as the reference electrode. The working electrolyte was a 0.5 M Na_2_SO_4_ aqueous solution, with a light exposure interval of 20 s, and the light source was a xenon lamp. The conditions for the EIS measurements were established with a frequency spectrum from 0.1 Hz to 100 kHz, utilizing a 200 W Xe lamp (BBZM-1, Bobei, Nanjing, China) as the source of visible light irradiation.

### 3.6. Photocatalytic Experiments

All adsorption tests were conducted in the absence of light and at room temperature.

(1) Sulfanilamide (SN) was utilized as the target pollutant, with 100 mg of catalyst introduced into a solution possessing an initial concentration of 10 mg·L^−1^. The shaking duration varied from 0 to 300 min to assess the adsorption capacity and selectivity. A 200 W Xe lamp source was employed during the experimental procedures, accompanied by a filter set at 420 nm. The concentration of residual sulfanilamide was quantified using UV–visible spectroscopy at a wavelength of λ = 258 nm, and the adsorption results were analyzed according to the relevant model. The removal rate of sulfanilamide (η, %) was calculated using Equation (1).

η% = (C_0_ − C_t_)/C_0_ = (A_0_ − A_t_)/A_0_ (1)

In the formula, C_0_ (mg·L^−1^) and C_1_ (mg·L^−1^) are the initial concentration and equilibrium concentration of sulfanilamide. A_0_ denotes the absorbance corresponding to the initial concentration of sulfanilamide.

(2) In the dye removal experiments, 100 mL of dye solution at varying concentrations was prepared, and 50 mg of catalyst was introduced to facilitate the degradation reaction on a magnetic stirrer. The wavelengths employed for the respective dyes were as follows: 464 nm for methyl orange (MO), 664 nm for methylene blue (MB), 554 nm for rhodamine B (RhB), and 617 nm for malachite green (MG). A 200 W Xe lamp acted as the irradiation light source in the experiments for photocatalytic degradation.

### 3.7. Determination of Point of Zero Charge

The point of zero charge of the sample was determined using the pH drift method. A series of 50 mL NaCl solutions (0.01 mol/L) were prepared as background electrolytes. The initial pH of each solution was adjusted in the range of 2.0 to 10.0 using 0.1 mol/L HCl and NaOH. An amount of 50 mg of sample was added to each solution. The suspensions were sealed and stirred at room temperature for 24 h to ensure equilibrium. Afterward, the final pH values of the solutions were measured.

### 3.8. DFT (Density Functional Theory) Calculations

The Castep module in Materials Studio (Materials Studio19.1) was used to simulate and calculate the band structure and density of state of the materials, based on first-principles DFT calculations. The Perdew–Burke–Ernzerhof (PBE) functional, combined with the generalized gradient approximation (GGA), was used to describe the ionic cores. The calculations were performed until the total energy converged to within 10^−5^ eV/atom, ensuring high accuracy of the optimization results.

## 4. Conclusions

B-doped BiOBr nanosheets with highly exposed surfaces were synthesized using water and acetic acid as solvents. Their photocatalytic degradation performance was tested with sulfanilamide (SN) and various organic dyes.

This study assessed BiOBr nanosheets synthesized under various hydrothermal conditions for sulfonamide degradation. The modified BiOBr sample, made with glacial acetic acid at 180 °C achieved a 60% removal rate, surpassing the WB180 sample from pure water. At a 10% boron (B) doping concentration, BiOBr exhibited optimal photocatalytic performance, with degradation rates of 99.2% for methyl orange (MO), 98.5% for malachite green (MG), 79.2% for methylene blue (MB), and 65.5% for rhodamine B (RhB). The degradation efficiency for sulfamethoxazole (SN) at 10 mg/L was 83.2%. Free radical capture experiments revealed that the superoxide radical (·O_2_^−^) is the main free radical in sulfamethoxazole photodegradation. The band gap energies of boron-doped BiOBr and pure BiOBr are 2.65 eV and 2.75 eV, respectively. The introduction of boron into the BiOBr framework increases the density of the electronic configuration and introduces an energy state within the band gap, facilitating the dissociation of charge carriers generated during illumination and boosting photocatalytic effectiveness in the degradation of pollutants.

## Figures and Tables

**Table 1:** Comparison of the performance of similar types of catalysts in the recent literature.

Photocatalysts	Light Source	Catalyst Dosage	Removal	Active Species	Ref.
1B-AB	200 W cold light xenon lamp	100 mg	6 h, 76%10 ppm SN 2 h, 92%20 ppm MG 2 h, 80%20 ppm MO	·O_2_^−^·OH	This work
3% Mo-BiOBr	300 W xenon lamp	300 mg	2 h, 80%10 ppm SN	·O_2_^−^·OH	[42]
BiOBr/chitin -Fe_3_O_4_	300 W xenon lamp	200 mg	3 h, 79%10 ppm SN	·O_2_^−^·OH	[43]
40% BiOCl/BiOBr	UV LED light	1000 mg	6 h, 93%10 ppm MB	h^+^·O_2_^−^	[44]
70-ZCS/BiOBr	500 W xenon lamp	100 mg	4 h, 99%20 ppm MB	·O_2_^−^·OH	[45]
10% Ag/BiOBr	Visible light	100 mg	5 h, 95%3 ppm MB	·O_2_^−^·OH	[46]
1:2 NS@MP	300 W xenon lamp	100 mg	5 h, 65%20 ppm BPA	·O_2_^−^	[47]
MoS_2_ QDs/BiOBr	500 W xenon lamp	40 mg	1 h, 99%20 ppm RhB 6 h, 87%10 ppm CIP	h^+^·O_2_^−^	[48]

## Data Availability

The raw/processed data required to reproduce these findings cannot be shared at this time due to technical limitations.

## Supplementary Materials

The following supporting information can be downloaded at: https://www.mdpi.com/article/10.3390/molecules30081735/s1, Figure S1. Adsorption curve of sulfonamides by 1B-AB over 60 minutes; Figure S2. Photolysis Experiment of SN, RhB, MO, MO and MG; Table S1. Structural characteristics of the sample.
