# Supplementary material for: Boron-Doped BiOBr Nanosheets with Enhanced Photocatalytic Activity for Sulfanilamide and Dyes
Source: Molecules. 2025 Apr 12;30(8):1735. doi: 10.3390/molecules30081735 (PMC12029954; doi:10.3390/molecules30081735)
Supplement: Supplementary file 1 [file molecules-30-01735-s001.zip › molecules-3560949-supplementary.pdf]

Boron-Doped BiOBr Nanosheets with Enhanced Photocatalytic Activity for Sulfanilamide and Dyes

Zimu Wei [1,2], Ying Wang [1], Zonghan Shao [1,2], Linkun Xie [1], Lianpeng Zhang [2], Kaimeng Xu [2], Xijuan Chai [1,2,*]

[1] Yunnan Key Laboratory of Wood Adhesive and Glued Products, Southwest Forestry University, Kunming 650224, China

[2] College of Material and Chemical Engineering, Southwest Forestry University, Kunming 650224, China

* Correspondence: xjchai@126.com

1. Figures

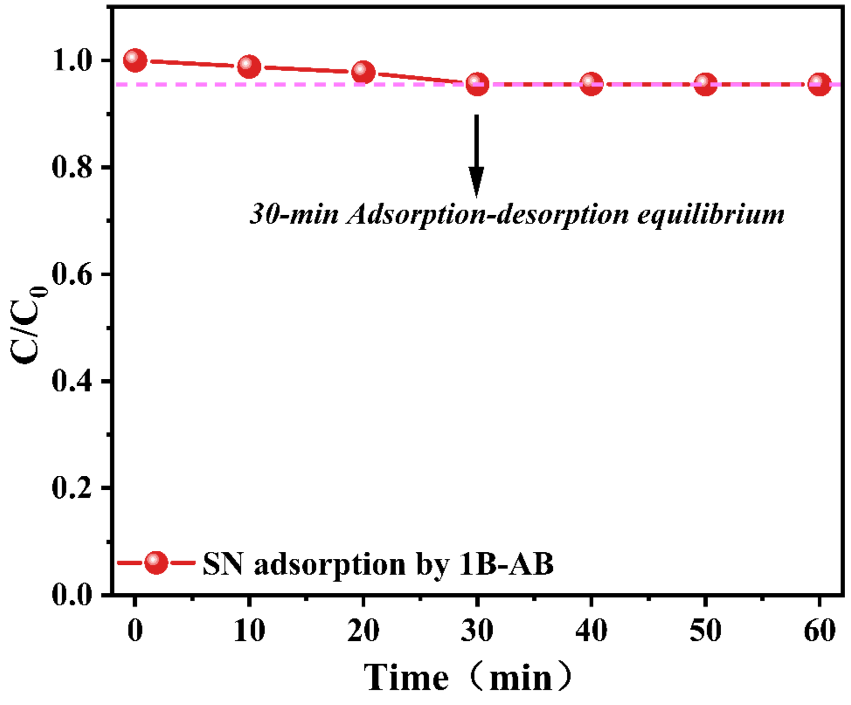

Figure S1. Adsorption curve of sulfonamides by 1B-AB over 60 minutes.

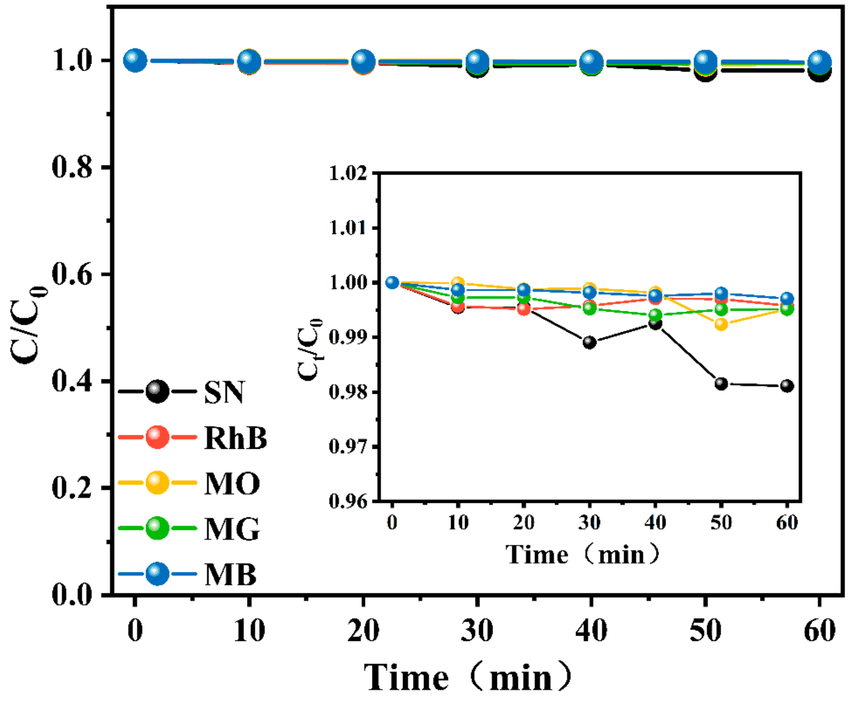

Figure S2. Photolysis Experiment of SN, RhB, MO, MO and MG.

2. Table

Table S1. Structural characteristics of the sample

sample	Surface area (m^2 / g)	Pore size (nm)
WB180	9.726	23.973
1B-AB	31.408	10.874
